# Synergistic Effects of Levodopa, Benserazide, and Nortriptyline on Behavioral Impairments and Brain Pathology in an Experimental Rat Model of Parkinson’s Disease

**DOI:** 10.1155/nri/9986180

**Published:** 2026-01-28

**Authors:** Maryam Ezzedin, Sam Zarbakhsh, Houman Parsaei, Ali Ghanbari, Abbas Ali Vafaei, Zohre Mohsenvand, Seyed Ali Seyedinia, Parnia Tarahomi, Manouchehr Safari

**Affiliations:** ^1^ Nervous System Stem Cells Research Center, Neuroscience Research Institute, Semnan University of Medical Sciences, Semnan, Iran, semums.ac.ir; ^2^ Research Center of Physiology, Neuroscience Research Institute, Semnan University of Medical Sciences, Semnan, Iran, semums.ac.ir

**Keywords:** anxiety, benserazide, levodopa, motor activity, nortriptyline, Parkinson’s disease

## Abstract

**Background:**

Parkinson’s disease (PD) is a progressive neurodegenerative disorder characterized by the degeneration of dopaminergic neurons in the substantia nigra pars compacta (SNpc). In addition to postural instability, rigidity, tremor, and bradykinesia, patients will experience depression and/or anxiety at any time during PD. Nortriptyline, as a dual reuptake inhibitor of norepinephrine and serotonin, inhibits alpha‐synuclein aggregation and may play an important role in improving the pathological effects of PD.

**Objective:**

This study investigated the effects of nortriptyline combined with L‐DOPA and benserazide on behavioral, histological, and biochemical changes in a rat model of PD. Methods. Forty‐nine rats were randomly assigned to seven groups. Except for the control and sham groups, five other groups underwent stereotactic surgery for the 6‐OHDA lesion. We performed a tail suspension swing test and an apomorphine‐induced rotation test after 1 week to confirm the PD model. After gradual treatment with three doses (5, 10, and 20 mg/kg) of nortriptyline combined with L‐DOPA and benserazide, the elevated plus‐maze test and open field test were performed to determine motor activities, anxiety, and depression. Tissue alterations were evaluated through Nissl staining, tyrosine hydroxylase immunohistochemistry, and Golgi–Cox staining, whereas oxidative stress levels were determined by analyzing malondialdehyde (MDA), superoxide dismutase (SOD), and total antioxidant capacity (TAC) markers.

**Results:**

Our results demonstrate that 10 mg/kg of nortriptyline in combination with L‐DOPA and benserazide significantly improved motor activity and reduced the anxiety‐ and depression‐like behaviors of PD. Histological findings also suggested a protective effect of nortriptyline on dopaminergic neurons in the SNpc. Furthermore, the findings from the antioxidant evaluation and the structure of CA1 hippocampal neurons indicated that a dosage of 10 mg/kg of nortriptyline might provide the greatest supportive benefit.

**Conclusion:**

Nortriptyline at 10 mg/kg offers a promising adjunctive therapy for alleviating both motor and nonmotor symptoms of PD. However, higher doses may induce anxiogenic effects, suggesting the need for careful dose optimization.

## 1. Introduction

Parkinson’s disease (PD) is a progressive neurodegenerative disorder characterized by bradykinesia, muscle rigidity, tremor, and balance and coordination disorders and affects approximately 1%–4% of people over 60 years of age [1–3]. In addition to motor symptoms, recent evidence highlights that PD is associated with a wide range of nonmotor symptoms, including depression, stress, anxiety, cognitive impairment, sleep disturbances, hyposmia, constipation, pain, and genitourinary problems, which may sometimes appear years before the onset of classical motor symptoms and can significantly contribute to the incidence and reduction in the quality of life of patients [4–6]. The World Health Organization has just released statistics showing that there are currently more than 6 million patients with PD worldwide. It is estimated that by 2030, more than 9 million people worldwide will suffer from this disease [7].

The main cause of this disease is the decrease in dopaminergic neurons in the pathway of the nigrostriatal system and the accumulation of ubiquitin and alpha‐synuclein (α‐syn) Lewy bodies in the substantia nigra pars compacta (SNpc) of the midbrain [8, 9].

6‐Hydroxydopamine (6‐OHDA) is an endogenous oxidant, known as a neurotoxin in the induction of PD, that can enter the dopaminergic neurons and, through increasing the level of ROS, can lead to cell death by apoptosis [10]. In addition to oxidative stress, 6‐OHDA induces neuronal death by essential molecular and cellular mechanisms associated with PD, including dysregulation of mitochondrial function, neuroinflammation, neurodegeneration, and disruption of the ubiquitin–proteasome system [11, 12]. Although the findings of some studies have demonstrated that 6‐OHDA does not result in any α‐syn aggregation in the brain [13, 14], there is definite evidence of the effective role of 6‐OHDA in increasing significantly α‐syn phosphorylation, which precedes neuronal apoptosis in the midbrain [10, 15].

L‐3,4‐Dihydroxyphenylalanine (L‐DOPA), also known as levodopa, is the precursor to the neurotransmitters dopamine, norepinephrine (NE), and epinephrine, which crosses the blood–brain barrier (BBB) and is converted into dopamine by the enzyme aromatic L‐amino acid decarboxylase; it increases dopamine levels in PD [16]. L‐DOPA has been the most popular treatment for PD for the past 40 years [17].

One problem with the systemic use of L‐DOPA is that the majority of L‐DOPA is converted to dopamine prior to reaching the brain, and as dopamine is unable to cross the BBB, this provides no therapeutic benefits for PD. Therefore, benserazide is used as an inhibitor of L‐DOPA decarboxylation in the treatment of PD [18]. Thanks to their pharmacodynamic and pharmacokinetic properties, L‐DOPA and benserazide are widely used in the management of PD, and their therapeutic benefits have been reported in many studies [7, 18, 19]. Anxiety in PD has been linked to neurodegeneration and dysfunction across multiple brain regions and neuronal circuits involved in emotion regulation, including the amygdala, prefrontal cortex, locus coeruleus, and serotonergic pathways. This widespread involvement of limbic and monoaminergic systems contributes to the high prevalence of anxiety symptoms in PD patients [20, 21]. Some animal studies in PD have shown that L‐DOPA may interfere with NE and serotonin (5‐HT) function in affect‐related brain structures and induce symptoms of anxiety and depression [22]. Recent preclinical studies suggest that chronic L‐DOPA administration may disrupt the balance of monoaminergic neurotransmission in emotion‐related brain regions. Specifically, L‐DOPA can increase dopamine synthesis at the expense of NE and 5‐HT, as it competes for the same enzymatic pathways and transport mechanisms. This competition may reduce NE and 5‐HT availability in limbic structures such as the amygdala and prefrontal cortex, leading to dysfunction in mood regulation circuits and the emergence of anxiety and depressive‐like behaviors [23, 24]. Consistent with this view, emerging pharmacological strategies in PD aim to restore the balance among dopaminergic, noradrenergic, and serotonergic systems, rather than focusing exclusively on dopamine replacement. Modulating serotonergic and noradrenergic transmission—rather than disrupting it—has shown potential in improving both motor complications (such as L‐DOPA‐induced dyskinesia) and nonmotor symptoms, including anxiety and depression, by compensating for the complex neurochemical imbalances associated with PD [25]. In this regard, Kamińska et al. evaluated the combined administration of L‐DOPA and desipramine (a tricyclic antidepressant) in a 6‐OHDA rat model of PD. Both drugs were administered intraperitoneally. Since L‐DOPA crosses the BBB and is converted to dopamine within the brain, the combined treatment significantly improved both motor and psychiatric performance in PD rats [26].

However, while L‐DOPA mainly targets dopaminergic deficits, additional neuroprotective strategies may further enhance therapeutic outcomes. Studies have shown that nortriptyline, a tricyclic antidepressant drug, inhibits neurotoxicity and aggregation of α‐syn through interaction with its monomeric form. Nortriptyline binds directly to α‐syn. It affects the ensemble of unfolded conformations to promote more rapidly diffusing, more expanded structures, thereby making the formation of the first oligomeric dimer less likely [27]. Anxiety, apathy, cognitive impairment, and dementia are symptoms of PD depression that are caused by the dysfunction of the limbic systems and monoaminergic systems. Evidence suggests that nortriptyline, as a dual reuptake inhibitor of NE and 5‐HT, is able to reduce PD depression [28].

It has been reported that nortriptyline indirectly reduces microglial activation, which is associated with PD, by inhibiting interleukin‐1 beta (IL1‐beta) and tumor necrosis factor‐alpha (TNF‐alpha) [29]. Additionally, the motor and nonmotor functions of patients with PD are affected due to significantly reduced levels of monoamines such as NE, 5‐HT, and dopamine. Studies have shown that nortriptyline inhibits the reuptake of NE and 5‐HT, increasing their synaptic concentrations [30, 31]. While it does not directly affect dopamine reuptake, elevated NE can enhance dopamine release through noradrenergic projections and receptor interactions in the basal ganglia and other brain regions implicated in motor control [32]. Furthermore, serotonergic modulation of dopaminergic neurons can influence dopamine synthesis and release, further impacting motor function [33]. These indirect effects on dopaminergic neurotransmission may underlie nortriptyline’s potential to alleviate motor symptoms in PD.

Although most evaluations focus on the antidepressant effect of nortriptyline in PD, evidence of the positive effect of this drug in improving motor activities has also been reported after stroke. [34]. As a result, evaluating the effect of nortriptyline on PD can help to discover other effects of this drug in PD and improve possible side effects of L‐DOPA, including anxiety and depression.

According to the mentioned background, in the present study, we investigated the effects of simultaneous administration of L‐DOPA, benserazide, and nortriptyline on the behavioral function and histological changes of the SNpc of the PD rat model.

## 2. Materials and Methods

### 2.1. Animals and Ethics

Forty‐nine adult male Wistar rats (weighing 200–250 g) were obtained from the laboratory animal breeding center, physiology research center of Semnan University of Medical Sciences. Animals were housed in standard polycarbonate cages (four rats per cage) with wood‐shaving bedding and environmental enrichment (cardboard tubes and nesting materials) to promote natural behaviors. Environmental conditions were maintained at 25°C ± 2°C, relative humidity of 40%–60%, and a 12/12 light/dark cycle. Standard laboratory chow and water were provided ad libitum. All experimental procedures, including animal surgeries, were performed in accordance with the National Institutes of Health (NIH) guidelines for the care and use of experimental animals and complied with the ARRIVE guidelines [35]. At the end of the experiments, rats were euthanized by an overdose of sodium pentobarbital (200 mg/kg, intraperitoneally), in accordance with the AVMA Guidelines for the Euthanasia of Animals (2020). The study protocol was approved by the Ethics and Research Committee of Semnan University of Medical Sciences (Approval code: IR.SEMUMS.REC.1399.199).

### 2.2. Experimental Protocol

The animals were randomly assigned into seven experimental groups (*n* = 7). Control group: Did not receive any intervention. Sham group: Animals underwent stereotaxic surgery without any intracerebral injection or pharmacological intervention. PD group: Received normal saline intraperitoneally after stereotaxic surgery. The first treatment group (PD + L): After confirming the induction of PD, they received L‐DOPA and benserazide (Sigma‐Aldrich, CAS Number: 14919‐77‐8) at doses of 10 and 2.5 mg/kg (ip, daily), respectively, for 2 weeks. The second treatment group (PD + N5): After confirming the induction of PD, they received nortriptyline (Sigma‐Aldrich, CAS Number: 894‐71‐3), L‐DOPA, and benserazide at doses of 5, 10, and 2.5 mg/kg (ip, daily), respectively, for 2 weeks. The third treatment group (PD + N10): After confirming the induction of PD, they received nortriptyline, L‐DOPA, and benserazide at doses of 10, 10, and 2.5 mg/kg (ip, daily), respectively, for 2 weeks. The fourth treatment group (PD + N20): After confirming the induction of PD, received nortriptyline, L‐DOPA, and benserazide at doses of 20, 10, and 2.5 mg/kg (ip, daily), respectively, for 2 weeks.

The doses of nortriptyline used in this study were selected based on previous preclinical studies evaluating the antidepressant and neuroprotective effects of tricyclic antidepressants in rodent models of PD and neurodegeneration [36]. Lower doses are commonly used to assess anxiolytic and antidepressant activity, while higher doses (10–20 mg/kg) have been employed to investigate potential dose–response relationships and therapeutic limits. We aimed to evaluate a low, moderate, and high dose to determine the optimal therapeutic window when co‐administered with L‐DOPA and benserazide.

### 2.3. Induction and Validation of PD Model Via 6‐OHDA Injection

The unilateral injection of neurotoxin 6‐OHDA in the SNpc was performed based on a previous study [8, 12, 26]. The anesthesia protocol consisted of ketamine and xylazine (100 and 20 mg/kg ip, respectively; Sigma‐Aldrich, USA) selected based on previous studies employing similar stereotaxic 6‐OHDA models of PD in rodents [8, 37–39]. A slightly higher dose of xylazine was selected to achieve the stable and deeper anesthesia required for the prolonged stereotaxic procedure, as reported in comparable studies. Throughout all surgical procedures, animals were carefully monitored for respiratory rate, reflexes, and recovery to minimize potential adverse effects related to the higher anesthetic dose. The rats were mounted in a stereotaxic frame, and the bregma was used for coordinates determined by the Paxinus and Watson atlas. The injection of a single dose of 6‐OHDA (16 μg/4 μL) into the left side (anterior, AP = −5.04 mm, lateral, ML = −2 mm, ventral, DV = −8.1 mm) was performed for 5 min at the rate of 1 μL/min with a 5‐μL Hamilton syringe. Following surgery, the animals were monitored until they were fully awake and then returned to their cages. Behavioral assessments were conducted at multiple time points to monitor the progression of PD‐like symptoms. Early motor asymmetry was observable at 1 week postsurgery, while stable and pronounced deficits were confirmed over the following 2 weeks using the tail suspension swing test (TSST) and apomorphine‐induced rotation test (AIRT).

### 2.4. Behavioral Testing

The motor imbalance was evaluated by the TSST and AIRT based on previous studies [8, 37, 40]. The elevated plus‐maze test (EPM) and open field test (OFT) were performed to measure motor activities, anxiety, and depression based on previous studies (9:00 a.m.–14:00 p.m. each day). All behavioral tests were performed by an observer blinded to the group.

#### 2.4.1. TSST

To determine asymmetrical motor behavior, we utilized the TSST, which has been described in the previous study [40]. Each rat was gently suspended by the base of its tail approximately 5 cm above a soft padded surface to prevent injury. The suspension lasted for 60 s, during which the number of full‐body swings (turns) was recorded, noting the direction of each swing (ipsilateral or contralateral to the lesion). The test was performed at four distinct time points: Day 1 (baseline, before stereotaxic surgery), Day 7 (to confirm Parkinsonian symptoms postsurgery), and Days 14 and 21 (to evaluate therapeutic effects). All assessments were conducted by an observer blinded to group allocation in a quiet, dimly lit room to reduce stress and environmental distractions.

#### 2.4.2. AIRT

The AIRT is a well‐established behavioral test used to assess dopamine depletion in rodents with unilateral 6‐OHDA lesions. The AIRT was conducted based on previous studies [8, 37]. The test was performed on Days 7 and 14 poststereotaxic surgery to evaluate the severity of dopaminergic neuron loss and to compare functional deficits with TSST results. Briefly, rats were given apomorphine hydrochloride (Sigma‐Aldrich, 2.5 mg/kg, ip) to induce rotational behavior. Following injection, each rat was immediately placed in a transparent cylindrical arena and video‐recorded for 30 min using a digital camera under standardized lighting conditions. Rotations were counted by three independent observers blinded to the treatment groups. Full 360° rotations toward the side contralateral to the lesion were recorded, and the net rotation score was calculated as the difference between contralateral and ipsilateral turns (contralateral minus ipsilateral rotations). This net rotation score serves as an indicator of dopaminergic imbalance in the striatum.

#### 2.4.3. OFT

The open field device consists of a black wooden box measuring 72 cm wide × 72 cm long and 4 black walls 50 cm high. The floor area was divided into 25 squares (18 × 18 cm) and defined by 2 areas, the center and the periphery. At the beginning of the experiment, each animal was placed in the central area, and the experiment started after 30 s of adaptation. The activity was recorded for 5 min under a low‐light environment (35–45 lux). EthoVision XT‐7 software (Noldus, Netherlands) is used for data monitoring and analysis. Cumulative duration in the central/corner zone, frequency of entries into the central zone, animal speed, and total distance traveled were measured.

#### 2.4.4. EPM

The EPM is a device made of wood and has four arms (50 cm long × 10 cm wide) in the form of a positive (+) sign and placed by bases at a height of 50 cm from the ground. The EPM apparatus had a central platform (10 × 10 cm) connected to two open corridors (0.5 cm high edge of glass) and two closed corridors (walls 40 cm high).

### 2.5. Histological Assay

Animals were deeply anesthetized with ketamine and xylazine (100 and 15 mg/kg, respectively). Afterward, the animals were transcardially perfused with normal saline, followed by 4% ice‐cold paraformaldehyde in phosphate buffer saline (PBS) (0.1 M, pH = 7.4). Brains were carefully removed, and after washing with normal saline, postfixed in 4% paraformaldehyde in 0.1 M PBS for 72 h at room temperature (RT). After that, the brain tissues were processed, embedded in paraffin blocks, and trimmed. Finally, 6–7 μm sections were prepared with a rotary microtome for histological evaluation based on the Paxinos and Watson atlas [41].

#### 2.5.1. Nissl Staining

Tissue staining was carried out using cresyl fast violet stain for histochemical demonstration of Nissl bodies in neurons [41, 42]. Briefly, brain sections were cleaned and then stained with 0.5% cresyl violet solution (24°C for 10 min). Finally, the sections were flushed and dehydrated with various ethanol concentrations (50%, 70%, 95%, and 100%), cleaned in xylene, and mounted on slides for histopathological assessments. Slides of six separate animals were observed with an Olympus microscope (Japan) and photographed (× 40 magnification). Neuron counting was done with ImageJ software.

#### 2.5.2. Tyrosine Hydroxylase (TH) Immunohistochemistry

The sections were deparaffinized and rehydrated. For quenching endogenous peroxidase activity, sections were incubated with 0.3% H2O2 (15 min). The sections were deparaffinized and rehydrated. For quenching endogenous peroxidase activity, sections were incubated with 0.3% H2O2 (15 min), followed by washing with distilled water and incubating them in citrate buffer 0.1 M pH 5.8 (10 min at 60°C–80°C). Background staining was blocked by 1.5% goat serum (15 min), followed by incubation by rabbit polyclonal antityrosine hydroxylase antibody (60 min) (1:1000; ab137869, Abcam, USA). After washing in PBS, the sections were incubated with the biotinylated secondary antibody (30 min at RT, 1:500, Cat. No. 65‐6140, Thermo Fisher Scientific, USA) followed by washes with PBS. Henceforward, horseradish peroxidase conjugate was applied (15 min at RT), DAB color development (5 min at RT), hematoxylin restaining (1 min at RT), dehydrated, cleared with xylene, and coverslipping were performed. TH‐positive cells were counted in six high‐magnification fields (× 400) with an Olympus microscope (Japan) [42].

#### 2.5.3. Golgi–Cox Staining

Modified Golgi–Cox staining was carried out following previous studies [43]. After fixation in 10% formalin for 24 h, the brains were incubated in Golgi–Cox solution (5 parts 5% potassium dichromate, 5 parts 5% mercuric chloride, 4 parts 5% potassium chromate, and 10 parts distilled water) for 50 days in darkness. Tissues were then dehydrated through graded ethanol (70%–100%), cleared with xylol, and embedded in paraffin at 50°C. A rotary microtome was used to cut coronal sections (25 μm) from −2.62 to −3.36 mm relative to Bregma using the Paxinos and Watson atlas. Sections were examined under a light microscope (Zeiss, Germany), focusing on the hippocampal region. Dendritic length and branching were quantified using ImageJ (Version 1.48). Three animals were randomly selected per group, with 10 sections analyzed per animal to calculate the mean dendritic parameters on both hemispheres of each section.

### 2.6. Biochemical Assay

#### 2.6.1. Malondialdehyde (MDA) Measurement

The assessment of MDA levels in hippocampal tissue was performed based on previous studies [44] and the thiobarbituric acid reactive substance (TBARS) method (Teb Pazhouhan Razi, Tehran, Iran). Briefly, following the manufacturer’s protocol, 100 mg of tissue was homogenized in ice‐cold 150 mM KCl containing butylated hydroxytoluene (BHT), then sonicated and centrifuged at 1600 × *g* (10 min, 4°C). Then supernatants were mixed with detergent and thiobarbituric acid (TBA) reagents and heated for 60 min at 95°C in a boiling water bath. Next, samples were cooled and centrifuged at 10, 000 × *g* (10 min, 4°C). The spectrophotometric absorbance was measured at 530–540 nm, and MDA concentrations were calculated from a 0–50 μM standard curve. The MDA level is expressed as μM per mg of tissue. All samples were analyzed in quadruplicate.

#### 2.6.2. Superoxide Dismutase (SOD) Activity Measurement

SOD activity in hippocampal tissue was measured using a commercial colorimetric kit (Nasdox™, Navand Salamat, Iran). Briefly, 100 mg of tissue was homogenized in 500 μL of ice‐cold lysis buffer (1:10 dilution in double‐distilled water), then centrifuged at 12,000 rpm for 5 min at 4°C. Following that, 50 μL of supernatant was added to the test wells, while 50 μL of deionized water was added to the control wells. Reagent 1 (pH 8.2) and Reagent 2 (prepared by mixing R2a and R2b, pH 7.4) were added sequentially. Next, the 96‐well plate was incubated at RT in the dark for 5 min. The spectrophotometric absorbance was recorded at 405 nm using a microplate reader. Finally, SOD activity was calculated based on the inhibition rate of pyrogallol autoxidation and expressed as U/mg protein, according to the kit instructions. All samples were analyzed in quadruplicate.

#### 2.6.3. Total Antioxidant Capacity (TAC) Measurement

TAC in hippocampal tissue was assessed following previous studies [44] utilizing a commercial assay kit (Naxifer™, Navand Salamat, Iran). The ferric‐reducing antioxidant power (FRAP) method was employed to assess the TAC, which reflects the ability of antioxidants to reduce Fe3+ to Fe2+. Briefly, 50–100 mg of tissue was homogenized in ice‐cold lysis buffer (1:10 w/v), followed by centrifugation at 10, 000 × *g* (10 min, 4°C). Next, a working solution was prepared according to the manufacturer’s instructions. In each well of a 96‐well microplate, 5 μL of supernatant or standard was added in quadruplicate, followed by 250 μL of the working reagent. After 5 min of incubation at RT, spectrophotometric absorbance was measured at 593 nm. TAC values were calculated from a standard curve (0–1.0 mM Fe^2+^ equivalents) using linear regression and expressed as mmol Fe^2+^ per mg of tissue protein.

### 2.7. Statistical Analysis

Data are presented as mean ± SEM. All statistical analyses were performed using SPSS Version 22.0. Before inferential testing, data distributions were assessed for normality using the Shapiro–Wilk test and for homogeneity of variances using Levene’s test. For datasets that met the assumptions of normality and homogeneity, one‐way analysis of variance (ANOVA) was used to compare groups, followed by Bonferroni’s post hoc test for multiple comparisons. For data that did not meet parametric assumptions, the nonparametric Kruskal–Wallis test followed by Dunn’s post hoc test was performed. Histopathological data that did not meet normality assumptions were analyzed using the Kruskal–Wallis test. In all analyses, differences were considered statistically significant at *p* ≤ 0.05.

## 3. Results

### 3.1. Behavioral Testing

#### 3.1.1. TSST

**FIGURE 1:**
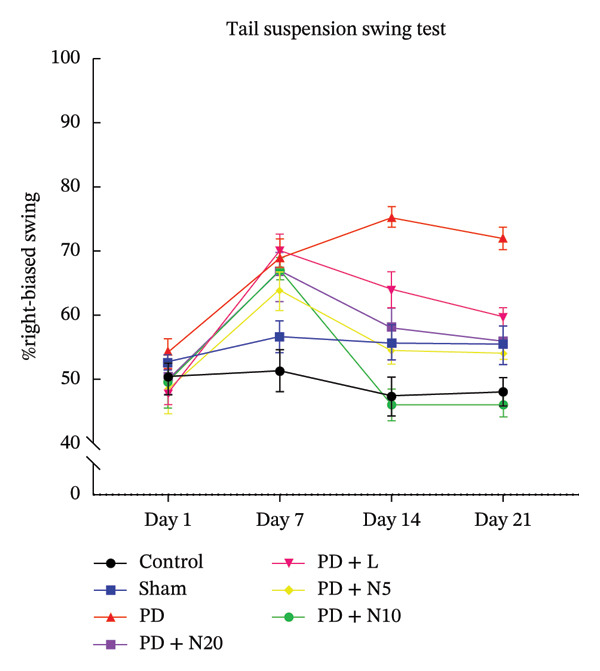
This figure illustrates the %right biased swing assessed by the TSST. Control (green), sham (black), PD (red), PD + L (yellow), PD + N5 (blue), PD + N10 (purple), PD + N20 (orange). Day 1 reports the presurgical testing. Day 7 represents the % right‐biased swing 7 days after stereotaxic surgery to validate the PD model. Days 14 and 21 represent the % right‐biased swing 7 and 14 days after the treatments. During 14 days, the %right bias swings increased to reach an average of about 75.33 swings in the PD group and then began to decrease slightly. The increase in %right‐biased swings confirmed the PD model in the other groups except the sham group on Day 7. Thereafter, the %right bias swings gradually decreased from Day 7 to Day 21, which was very significant in the PD + N10 group (*p* < 0.001).

The first TSST was performed before performing stereotaxic surgery and any invasive procedure. Our result showed no significant difference between the mean values of the first TSST between the control and the other six groups (F: 2.98, DFn: 6, DFd: 35, *p* > 0.05). The second TSST was performed 7 days after stereotaxic surgery. There was a significant difference in the mean TSST values between the control group and the other six groups, as well as the sham group and the other groups (F: 25.25, DFn: 6, DFd: 35, *p* < 0.0001). In other cases, no significant relationship was observed. After starting the treatment, the TSST test was done 1 week and 2 weeks later. The results showed that there is a significant difference in the percentage of right bias swing between the studied groups after treatment (F: 91.36, DFn: 6, DFd: 35, *p* < 0.0001). In the following, post hoc statistical analyses have shown that PD + N10 indicated the most significant decrease of %Right biased swing compared to other groups during the 3rd stage of the TSST, which was performed 1 week after treatment (*p* < 0.0001). Interestingly, there was no significant difference between the control and PD + N10 groups (*p* = 0.971). In the fourth stage of the TSST, a significant difference was seen between the PD group and the other groups (F: 73.42, DFn: 6, DFd: 35, *p* < 0.0001). The PD + N10 group had the least %Right biased swing compared to other groups (*p* < 0.0001), similar to the control group, and there was no significant difference (*p* = 0.788). There was no significant difference between the sham, PD + L, PD + N5, and PD + N20 groups at this stage (*p* > 0.05) (Figure 1).

#### 3.1.2. AIRT

**FIGURE 2:**
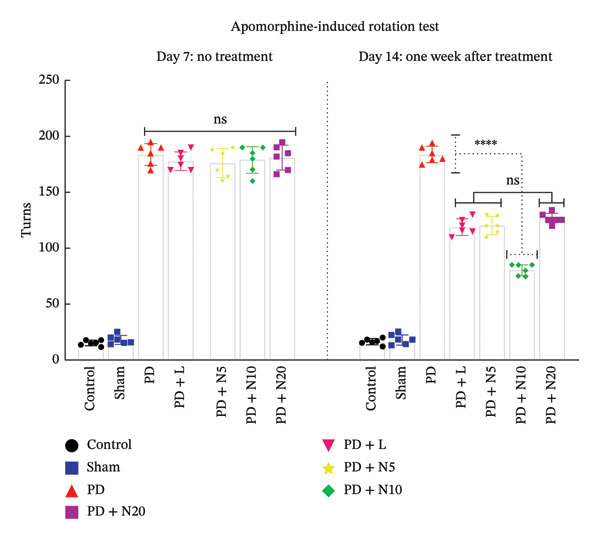
This figure illustrates the number of rat turns during 30 min assessed by the AIRT. ns: *p* > 0.05, ^∗∗∗∗^ *p* < 0.0001.

The AIRT was performed to monitor the motor impairment induced by the 6‐OHDA lesion and evaluate the preservation or restoration of dopaminergic neuron function. The results of our study showed that seven days after induction of Parkinson’s models, all rats treated with 6‐OHDA had signs and symptoms consistent with PD (F: 427.4, DFn: 6, DFd: 35, *p* < 0.0001). The difference between the sham and control groups was not significant (*p* :  0.999). Also, the difference between the 6‐OHDA receiving groups was not significant (*p* > 0.05). On the 14th day, there was a significant difference between the 6‐OHDA receiving groups and the healthy groups (F: 624.5, DFn: 6, DFd: 35, *p* < 0.0001). In addition, it has been demonstrated that there was a significant decrease in the rat contralateral net turn on the 14th day of the PD + N10 group compared to other groups (*p* < 0.001). Compared to the PD group, there was also a significant decrease in the number of contralateral net turns for the PD + L, PD + N5, and PD + N20 groups, but these three groups have not shown a significant difference together (*p* < 0.05 and *p* > 0.05, respectively) (Figure 2).

#### 3.1.3. OFT

The results of the OFT showed that the PD + N10 group (*n* = 6) spent less time in the corner of the open field compared with the PD group (*n* = 6) and PD + L (median for PD + N10 group vs. PD group and PD + L: 275/14 ± 14/4 vs. 293/05 ± 4/9 and 293/50 ± 2/5 s; *p* :  0.023 and *p* :  0.019, respectively) and spent a long time in the central area (24/90 ± 14/4 vs. 6/98 ± 4/8 and 6/53 ± 2/5 s; *p* = 0.023 and *p* :  0.018, respectively). There was no significant relationship between the PD and other groups in the comparison of the average time spent by the animal in the central/corner area (*p* > 0.05) (Table 1). It can be concluded that anxiety behaviors in the PD + N10 have significantly decreased compared to the PD group and PD + L.

**TABLE 1:** Behavioral results of the open field test.

Group	Number	Time spent in center	Time spent in corner	Enter to the center (n)	Speed of the animal (m/s)	Distance traveled (cm)
Control	5	13.40 ± 6.7	286.64 ± 6.8	13.80 ± 3.70	11.6 ± 2.88	4040.18 ± 728.41^#^
Sham	6	13.18 ± 4.6	286.86 ± 4.4	14.67 ± 6.43	11.66 ± 2.59	3254.13 ± 867.72
PD	6	6.98 ± 4.89	293.05 ± 4.9	7.83 ± 3.71	6.33 ± 3.26	2707.67 ± 515.26
PD + L	6	6.53 ± 2.5	293.50 ± 2.5	6.83 ± 3.48	8.83 ± 2.48	2260.62 ± 207.99
PD + N5	6	9.74 ± 4.94	290.29 ± 4.9	9.83 ± 4.07	8.16 ± 3.65	3112.63 ± 1139.64
PD + N10	6	24.90 ± 14.4^∗^	275.14 ± 14.4^∗∗^	23.66 ± 8.29^∗∗∗^	17.17 ± 3.76^∗∗∗∗^	3398.94 ± 444.35
PD + N20	5	19.86 ± 16.29	280.17 ± 16.3	13.40 ± 5.7	10.2 ± 3.03	2987.38 ± 575.70

^∗^Compared with PD and PD + L groups (*p* :  0.023 and *p* :  0.018, respectively).

^∗∗^Compared with PD and PD + L groups (*p* :  0.023 and *p* :  0.019, respectively).

^∗∗∗^Compared with PD, PD + L, PD + N5 and PD + N20 groups (*p* :  0.002, *p* < 0.0001, *p* :  0.001 and *p* :  0.04, respectively).

^∗∗∗∗^Compared with PD, PD + L, PD + N5 and PD + N20 groups (*p* < 0.0001, *p* :  0.001, *p* :  0.004 and *p* :  0.01, respectively).

^#^Compared with PD and PD + L groups (*p* :  0.04 and *p* :  0.003, respectively).

In addition, findings showed that the average distance traveled by rats in the control group was not significantly different from those treated with nortriptyline. Still, the PD and PD + L groups traveled significantly less distance than the Control group (*p* :  0.04, and *p* :  0.003, respectively) (Table 1).

Follow‐up statistical tests showed that the average number of times the animal entered the center in the PD + N10 group is longer than in the PD + N5 and PD + N20 groups (*p* :  0.001 and *p* :  0.04, respectively), similar to movement speed (*p* :  0.004 and *p* :  0.01, respectively). It can be concluded that 10 mg/kg of nortriptyline treatment improves motor activity in PD rats (Table 1).

#### 3.1.4. EPM Test

Representative tracings of the movement of the rats in the EPM test are shown in Table 2.

**TABLE 2:** Behavioral results of the elevated plus‐maze test.

Group	Number	Time spent in open arms (sec)	Enter into the open arms (n)
Control	5	87.40 ± 11.5	9.2 ± 1.3
Sham	6	67.66 ± 7.1	5.33 ± 1.2
PD	6	27.33 ± 10.3	2.83 ± 1.1
PD + L	6	39.67 ± 6.4	3.50 ± 0.5
PD + N5	6	67.5 ± 4.3^∗^	6.33 ± 1.3^∗^
PD + N10	6	80.67 ± 16.8^#^	7.16 ± 1.1^#^
PD + N20	5	45.80 ± 12.3	4.00 ± 1.0

^∗^Compared with PD, PD + L and PD + N20 groups (*p* < 0.0001, *p* :  0.001, and *p* :  0.02, respectively).

^#^Compared with PD, PD + L and PD + N20 groups (*p* < 0.0001, *p* < 0.0001, and *p* < 0.0001, respectively).

There was a significant difference in the time spent in the open arms between different groups (F: 21.39, DFn: 6, DFd: 33, *p* < 0.0001). The PD + N10 (*n* = 6) and the PD + N5 (*n* = 6) groups spent a long time in the open arms compared with the PD group (*n* = 6) (median for PD + N10 and the PD + N5 groups vs. PD group: 80/67 ± 16/8 and 67/5 ± 4/3 vs. 27/33 ± 10/3 s; *p* < 0.001 and *p* < 0.001, respectively). Also, the rats in the PD + N10 and PD + N5 groups spent more time in the open arm than in the PD + N20 group (median for PD + N10 and the PD + N5 groups vs. PD + N20 group: 80/67 ± 16/8 and 67/5 ± 4/3 vs. 45/80 ± 12/3 s; *p* < 0.001 and *p* :  0.02, respectively). There was no significant difference between the control, sham, PD + N5, and PD + N10 groups (*p* > 0.05).

The number of entering the open arms was also significantly different in different groups (F: 23.79, DFn: 6, DFd: 33, *p* < 0.0001). The number of entering the open arms in the PD + N10 and the PD + N5 groups was significantly higher than the PD and PD + N20 groups (median for PD + N10 and the PD + N5 groups vs. PD and PD + N20 groups: 7/16 ± 1/1 and 6/33 ± 1/3 vs. 2/83 ± 1/1 and 4.0 ± 1/0, respectively) (Table 2). There was no significant difference between the control and PD + N10 groups (*p* :  0.054).

### 3.2. Histological Assay

#### 3.2.1. Nissl Staining

**FIGURE 3:**
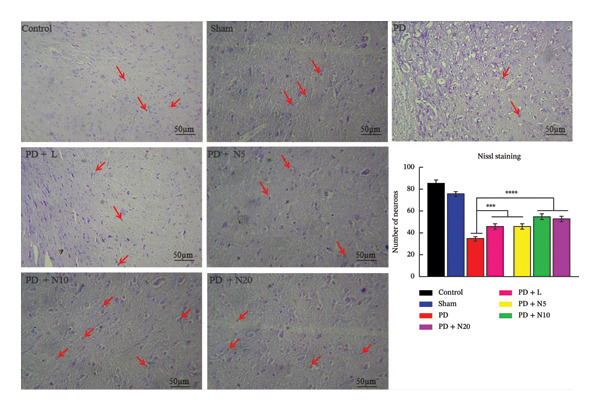
This figure illustrates the Nissl stain analysis to assess SNpc neuron damage. Scale bar = 50 μm. Data represent the mean ± SEM of the number of surviving neurons per section. Arrows indicate representative neurons. All nortriptyline groups (PD + N5, N10, N20) received co‐administration of L‐DOPA and benserazide ^∗∗∗^ *p* < 0.001, ^∗∗∗∗^ *p* < 0.0001.

According to Nissl staining images, the best neuroprotective effect was done in the PD + N10 group. The animals that received a combination of nortriptyline, L‐DOPA, and benserazide experienced the neuroprotective effect over the experiment period. Statistical analysis also showed that the PD + L, PD + N5, PD + N10, and PD + N20 groups have significantly more neurons compared to the PD group (median ± SD: 46.2 ± 1.35, 45.2 ± 1.42, 55.2 ± 1.42, and 55.0 ± 1.3 vs. 35.2 ± 2.0 *n*; *p* < 0.001, respectively). The beneficial roles of nortriptyline in combination with L‐DOPA and benserazide in the behavioral functions of PD‐treated rats led us to hypothesize that nortriptyline may protect dopaminergic neurons from 6‐OHDA‐induced apoptosis (Figure 3).

To evaluate this hypothesis, we first analyzed whether nortriptyline treatment prevents general neuron loss induced by 6‐OHDA, and Nissl staining was performed for this aim. The results showed that 6‐OHDA injection resulted in a great loss of neurons. The application of nortriptyline combined with L‐DOPA and benserazide almost significantly reversed this decrease (Figure 3).

#### 3.2.2. TH Immunohistochemistry

**FIGURE 4:**
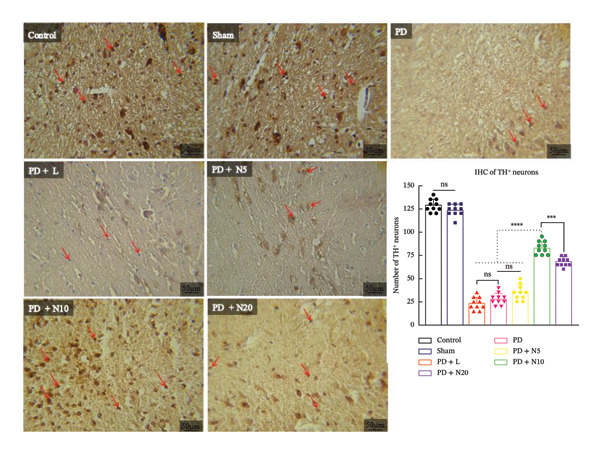
This figure illustrates an immunohistochemical staining analysis to detect TH expression in the SNpc neurons. Scale bar = 50 μm. Representative IHC images of TH^+^ neurons in the substantia nigra of different experimental groups. Brown DAB staining indicates TH‐positive dopaminergic neurons, which are highlighted with arrows in the images. The bar graph shows quantitative analysis of TH^+^ neurons (mean ± SEM). All nortriptyline groups (PD + N5, N10, N20) received co‐administration of L‐DOPA and benserazide. ^∗^ *p* < 0.05, ^∗∗^ *p* < 0.01, ^∗∗∗^ *p* < 0.001, ^∗∗∗∗^ *p* < 0.0001; ns: not significant.

To confirm Nissl staining findings, we used the TH immunohistochemistry method. We analyzed TH expression in the SNpc, as dopaminergic neurons are the main tissues for producing TH in vivo. As shown in Figure 4, 6‐OHDA induced a marked decrease in the TH‐positive neurons. As expected, the administration of nortriptyline combined with L‐DOPA and benserazide treatment prevented this decrease. Immunohistochemical analysis of TH‐positive neurons revealed a significant reduction in the number of dopaminergic neurons in the PD group compared to the control and sham groups (*p* < 0.0001). Treatment with L‐DOPA (PD + L) did not significantly restore TH expression. However, administration of the compound nortriptyline at higher doses. PD + N10 and PD + N20 groups significantly increased the number of TH‐positive neurons compared to the untreated PD group (*p* < 0.001 and *p* < 0.0001, respectively). Surprisingly, the PD + N10 group demonstrated a remarkable ability to significantly inhibit the degeneration of dopaminergic neurons compared to the other treatment groups (*p* < 0.0001). These findings suggest a dose‐dependent neuroprotective or neurorestorative effect of the compound nortriptyline on dopaminergic neurons in the SNpc.

#### 3.2.3. Golgi–Cox Staining

**FIGURE 5:**
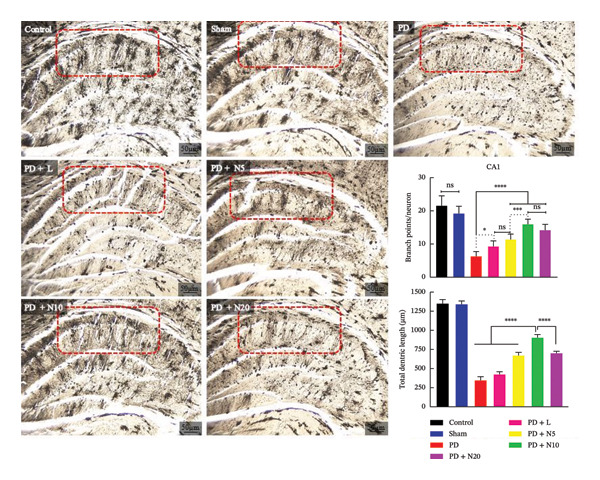
Golgi–Cox staining of hippocampal CA1 pyramidal neurons in different experimental groups. Scale bar = 50 μm. Besides that, quantitative analysis of dendritic branching and total dendritic length has been reported. The CA1 region is highlighted with a dashed rectangle. Data are expressed as mean ± SEM. ^∗^ *p* < 0.05, ^∗∗^ *p* < 0.01, ^∗∗∗^ *p* < 0.001, ^∗∗∗∗^ *p* < 0.0001; ns: not significant.

To assess neuronal morphology, Golgi–Cox staining was performed to evaluate dendritic branching and total dendritic length in the CA1 region of the hippocampus. Representative micrographs showed a clear reduction in dendritic complexity in the PD group compared to the control and sham groups. Quantitative analysis revealed a significant decrease in dendritic branch number in the PD group compared to the control (^∗∗∗∗^ *p* < 0.0001) and sham groups (^∗∗∗∗^ *p* < 0.0001). Treatment with nortriptyline significantly increased dendritic branching compared to the PD group (^∗∗∗^ *p* < 0.001), while the PD + L group showed moderate improvements. Although the groups treated with nortriptyline experienced a significant increase in the quantity of dendritic branches compared to the PD group, the PD + N5 group did not display a significant difference from the PD + L group (ns: *p* = 0.2321) (Figure 5).

Similarly, total dendritic length was significantly reduced in the PD group compared to the control and sham groups (^∗∗∗∗^ *p* < 0.0001). Among the treatment groups, PD + N10 showed the most substantial increase in dendritic length (^∗∗∗∗^ *p* < 0.0001 vs. PD), reaching levels close to the control group. The PD + N5 and PD + N20 groups also showed significant improvements compared to PD (^∗∗∗∗^ *p* < 0.0001 and ^∗∗∗∗^ *p* < 0.0001, respectively), while there was no significant difference observed between the two groups (ns: *p* = 0.6042).

These findings indicate that PD causes significant structural damage to the CA1 neurons in the hippocampus, and treatment with N10 offers the most effective neuroprotective advantages regarding dendritic branching and total dendritic length (Figure 5).

### 3.3. Biochemical Assay

**FIGURE 6:**
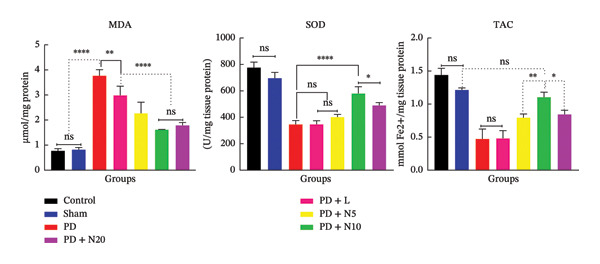
Oxidative stress markers in the hippocampal tissue of experimental groups. Bar graphs show the levels of malondialdehyde (MDA), superoxide dismutase (SOD) activity, and total antioxidant capacity (TAC) across different groups. Data are expressed as mean ± SEM. ^∗^ *p* < 0.05, ^∗∗^ *p* < 0.01, ^∗∗∗^ *p* < 0.001, ^∗∗∗∗^ *p* < 0.0001; ns: not significant.

The antioxidant profile was evaluated by measuring MDA, SOD, and TAC levels in different experimental groups. MDA levels were significantly elevated in the PD group compared to the sham group (^∗∗∗∗^ *p* < 0.0001). Treatment with L (PD + L) and N (PD + N5, PD + N10, and PD + N20) resulted in a significant reduction in MDA levels compared to the sham group (^∗∗^ *p* < 0.01 and ^∗∗∗∗^ *p* < 0.0001, respectively). While the PD + N10 group exhibited the most significant decrease in MDA levels among the nortriptyline‐treated groups, there was no statistically significant difference between the PD + N10 group and the PD + N20 group (ns: *p* = 0.9458) (Figure 6).

SOD and TAC activity was significantly reduced in the PD group compared to the control (^∗∗∗∗^ *p* < 0.0001), suggesting the presence of oxidative stress. Treatment with N10 (PD + N10) notably restored SOD and TAC levels compared to the PD group (^∗∗∗∗^ *p* < 0.0001); MDA levels in the PD + L and PD + N5 groups did not show significant changes (ns, *p* > 0.9999and *p* = 0.3612, respectively); the TAC levels also did not show a significant difference between the PD + L and PD groups (ns, *p* > 0.9999). Interestingly, the TAC level in the PD + N10 group increased considerably when compared to the other treatment groups and showed no significant difference from the sham group (ns, *p* = 0.5743). The findings suggest that the PD + N10 group had the most significant effect on enhancing antioxidant defenses by increasing SOD and TAC levels while decreasing MDA levels in comparison to the other treatment groups (Figure 6).

## 4. Discussion

PD is the second most common neurological disease after Alzheimer’s disease and is associated with the loss of dopaminergic neurons in the SNpc area of the midbrain [45]. A combination of L‐DOPA and benserazide has been the standard treatment for this disease until now [46]. The effect of nortriptyline at 3 different doses combined with L‐DOPA and benserazide in the Parkinson’s rat model has been investigated in this study.

As mentioned above, our study demonstrated that motor imbalance behavioral symptoms associated with PD in TSST and AIRT have been significantly improved by combination treatment with 10 mg/kg nortriptyline. The results concerning the behavioral tests were validated by the earlier preprint published by our research team [12, 47], and the replication of these tests has been reaffirmed in the current study. Anxiety behaviors have significantly decreased in the PD + N10. Furthermore, histological studies have shown that the PD + N10 group has a neuroprotective effect. However, the aforementioned findings did not show a significant improvement in the motor activity in PD rats during OFT and EPM.

In our study, the rats that received the 6‐OHDA showed motor imbalance and asymmetry symptoms after 1 week. Our finding is also consistent with studies that reported SNpc lesions and depletion of dopaminergic neurons caused by unilateral injection of 6‐OHDA into the SNpc [40, 48]. Dopamine is a widely used antiparkinsonian drug, but one of the drug’s side effects is anxiety and stress [22, 23]. However, it may be caused by PD depression or dysfunction of the limbic system and neurotransmitters such as NE and 5‐HT [28, 49]. Recently, studies have evaluated the effects of simultaneous antidepressant and L‐DOPA therapy in the treatment of PD. Kamińska et al. confirmed that combined treatment of desipramine + L‐DOPA could co‐modulate noradrenergic and dopaminergic transmission and positively influence motor and psychiatric functions in unilateral 6‐OHDA lesions [26].

Our results showed that the combination treatment of nortriptyline and dopamine can prevent the occurrence of depression and anxiety symptoms. Dobkin et al. confirmed the positive effects of nortriptyline on depression caused by PD in a randomized placebo‐controlled trial study [50]. Nortriptyline is a dual reuptake inhibitor, and it may exert an antidepressant effect by inhibiting the reuptake of 5‐HT and noradrenaline. In the present study, we observed that the nortriptyline treatment groups spent significantly more time in the open arm of the EPM, and the number of entries into the open arm was greater in these groups. The results demonstrated that the combination of nortriptyline with levodopa and benserazide improved depression and anxiety behavior in PD rats.

Collier et al. have shown that nortriptyline is beneficial in reducing the pathological aggregation of α‐syn in the in vitro and in vivo model of PD, which leads to the improvement of PD depression symptoms. The stereological count of TH‐positive and Nissl‐stained neurons in SNpc was not significant compared to the control group [27]. Our TH immunohistochemistry result revealed a significant loss of dopaminergic neurons in the SNpc of the PD group. Treatment with higher doses of compound nortriptyline (PD + N10 and PD + N20) markedly restored TH^+^ neuron numbers, suggesting a neuroprotective or restorative effect. Notably, the significant improvement observed in the PD + N10 group was evident in both Nissl and TH staining. The parallel findings from these two histological methods strongly support the neuronal survival‐promoting potential of nortriptyline in the PD model.

The effect of three doses of nortriptyline (5, 10, and 20 mg/kg) in combination with L‐DOPA and benserazide has been evaluated in our study. The dramatic effect of the 10 mg/kg of nortriptyline was remarkable (PD + N10). We found that PD + N10 rats spent significantly more time in the center of the open field than other groups and had more exploratory behavior. In histological evaluations, 10 mg/kg of nortriptyline has been shown to have a significantly beneficial effect in protecting dopaminergic neurons of the SNpc. As a result, 10 mg/kg of nortriptyline is an effective dose to improve depression, anxiety behaviors, and motor activity.

Regarding side effects, no obvious adverse behavioral effects (e.g., sedation, hyperactivity, aggression, weight loss, or mortality) were observed in the animals during the 14‐day treatment period. However, behavioral assessments such as the OFT and EPM tests suggested that the highest dose (20 mg/kg) might reduce exploratory behavior and potentially induce anxiety‐like effects, indicating a possible anxiogenic response at higher doses. Bis‐Humbert et al. demonstrated that increasing the dose of nortriptyline had the opposite effect and could exhibit anxiogenic behavior in a dose‐dependent manner by reducing the exploratory time in the open field [36]. In our behavioral evaluation, rats treated with 5 and 10 mg/kg of nortriptyline showed higher exploratory activity in the EPM, as well as an OFT. Furthermore, the time spent in the center and entering the center (*n*) of the open field and the open arm of the EPM is longer than rats treated with 20 mg/kg of nortriptyline. Thus, more caution should be taken when administering different doses of nortriptyline in the treatment of depression and anxiety caused by PD.

The present study investigated oxidative stress markers and dendritic morphology in the hippocampus of a 6‐OHDA rat model of PD. Our findings revealed that PD induction significantly elevated MDA levels while decreasing SOD activity and TAC, indicating a pronounced oxidative imbalance in the hippocampal tissue. Among the treatment groups, the N10 dose was the most effective in reversing these changes, demonstrating its potential neuroprotective and antioxidant properties. In parallel observation, Golgi–Cox staining of the CA1 subregion revealed marked structural alterations in the dendritic structure in the PD group. A significant reduction in both dendritic branching and total dendritic length was observed compared to control and sham animals. These findings align with previous studies reporting that the CA1 region of the hippocampus is particularly susceptible to neurodegeneration and oxidative damage in PD models [51]. Administration of N10 substantially preserved the dendritic structure, restoring both branch complexity and length, which suggests its efficacy in mitigating PD‐related neuronal damage.

The CA1 region serves as the primary output point of the hippocampus, relaying processed information to cortical areas, and plays a crucial role in cognitive processing, memory formation, and emotional regulation [52]. Notably, recent research has emphasized that CA1 is not only affected in motor‐related pathology but also in nonmotor symptoms such as anxiety and stress, which are prevalent in PD patients [53]. In our study, the observation that antioxidant imbalance co‐occurred with dendritic degeneration in CA1 supports the hypothesis that oxidative stress contributes directly to structural and functional impairments in this region. This is consistent with the findings of Kim et al., who demonstrated dendritic atrophy in CA1 pyramidal neurons of 6‐OHDA‐lesioned rats, suggesting a correlation with PD‐related behavioral deficits [51]. The CA1 region of the hippocampus appears particularly vulnerable to oxidative stress, which may help explain the significant dendritic degeneration observed in our PD model. This heightened vulnerability may result from the combination of elevated synaptic activity and an inherently low capacity for antioxidant defense mechanisms in CA1 neurons, making them highly susceptible to oxidative stress‐induced structural degeneration in PD [54].

One limitation of the present study is the absence of desipramine pretreatment before 6‐OHDA administration. While our goal was to model the broader neurodegenerative profile of PD, including noradrenergic involvement, the lack of desipramine might allow partial uptake of 6‐OHDA by noradrenergic neurons. Although the localized stereotaxic injection minimizes off‐target diffusion, we cannot completely rule out minor effects on noradrenergic systems. Future studies combining desipramine pretreatment and more targeted lesion approaches may help to isolate the specific contributions of dopaminergic versus noradrenergic pathways, particularly in relation to nonmotor symptoms.

Another limitation is that this study was performed in a single neurotoxin‐induced model, which may not fully represent the progressive and multifactorial nature of human PD. In addition, while oxidative stress markers were biochemically assessed, the underlying molecular and genetic pathways involved in nortriptyline’s neuroprotective effects remain to be clarified. Future studies employing gene expression profiling, signaling pathway analysis, and long‐term evaluations in both sexes could provide deeper insights into the mechanisms and translational potential of nortriptyline co‐administration with L‐DOPA and benserazide.

## 5. Conclusion

In conclusion, nortriptyline demonstrates promising therapeutic potential in a rat model of PD, not only alleviating levodopa‐induced side effects but also enhancing motor performance and mood‐related behaviors. Importantly, our findings reveal a dose‐dependent effect, where low doses exert beneficial outcomes while higher doses may lead to adverse behavioral responses. Furthermore, the observed improvements in antioxidant status and dendritic morphology—particularly within the CA1 region of the hippocampus—highlight the critical role of oxidative stress and structural integrity in both motor and nonmotor manifestations of PD. These results support the notion that targeting neuroprotective pathways alongside traditional dopaminergic therapies may offer a more comprehensive approach for PD treatment.

## Author Contributions

Manouchehr Safari and Sam Zarbakhsh: conception and design, supervision and verification, and project administration. Ali Ghanbari: stereotaxic surgery and methodology. Abbas Ali Vafaei: behavioral tests, methodology, data collection, software, and analysis. Houman Parsaei, Maryam Ezzedin, Seyed Ali Seyedinia, Parnia Tarahomi, and Zohre Mohsenvand: behavioral tests, stereotaxic surgery, data collection and analysis, histological assay, and writing–original draft. All authors agree to be accountable for the research presented.

## Funding

This study was supported by Semnan University of Medical Sciences, Semnan, Iran, a‐10‐114‐14, 2020.

## Disclosure

All authors read and approved the final manuscript.

## Conflicts of Interest

The authors declare no conflicts of interest.

## Supporting Information

All experimental procedures were conducted in accordance with the National Institute of Health (NIH) guidelines for the care and use of experimental animals, as well as in compliance with the ARRIVE guidelines, as detailed in the attached Supporting Information, “The ARRIVE guidelines 2.0: author checklist.”

Supporting file 1: Graphical abstract of the study.

Supporting file 2: Highlights of the manuscript.

## Supporting information


**Supporting Information** Additional supporting information can be found online in the Supporting Information section.

## Data Availability

The data that support the findings of this study are available from the corresponding author upon reasonable request.

## References

[1] Lockhart T. , Frames C. , Olson M. , Moon S. H. , Peterson D. , and Lieberman A. , Effects of Protective Step Training on Proactive and Reactive Motor Adaptations in Parkinson’s Disease Patients, Frontiers in Neurology. (2023) 14, 10.3389/fneur.2023.1211441. PMC10642212 37965161

[2] Zarbakhsh S. , Safari M. , Aldaghi M. R. et al., Irisin Protects the Substantia Nigra Dopaminergic Neurons in the Rat Model of Parkinson’s Disease, Iranian Journal of Basic Medical Sciences. (2019) 22, no. 7, 722–728, 10.22038/ijbms.2019.33444.7987, 2-s2.0-85073040306. 32373292 PMC7196356

[3] Peng S. , Wang C. , Ma J. et al., Polypeptide Protects Dopaminergic Neurons From Apoptosis in Parkinson’s Disease Models Both In Vitro and In Vivo, British Journal of Pharmacology. (2018) 175, no. 4, 631–643, 10.1111/bph.14110, 2-s2.0-85040986350. 29181847 PMC5786457

[4] Chaudhuri K. R. , Healy D. G. , and Schapira A. H. V. , Non-Motor Symptoms of Parkinson’s Disease: Diagnosis and Management, The Lancet Neurology. (2006) 5, no. 3, 235–245, 10.1016/S1474-4422(06)70373-8, 2-s2.0-32544432029. 16488379

[5] Postuma R. B. and Berg D. , Advances in Markers of Prodromal Parkinson Disease, Nature Reviews Neurology. (2016) 12, no. 11, 622–634, 10.1038/nrneurol.2016.152, 2-s2.0-84994126501. 27786242

[6] Schapira A. H. V. , Chaudhuri K. R. , and Jenner P. , Non-Motor Features of Parkinson Disease, Nature Reviews Neuroscience. (2017) 18, no. 7, 435–450, 10.1038/nrn.2017.62, 2-s2.0-85021141305. 28592904

[7] Yang Y. , Gao F. , Gao L. , and Miao J. , Effects of Rasagiline Combined With Levodopa and Benserazide Hydrochloride on Motor Function and Homocysteine and IGF-1 Levels in Elderly Patients With Parkinson’s Disease, BMC Neurology. (2023) 23, no. 1, 10.1186/s12883-023-03411-3. PMC10557206 37803329

[8] Ghahari L. , Safari M. , Jaberi K. R. , Jafari B. , Safari K. , and Madadian M. , Mesenchymal Stem Cells With Granulocyte Colony-Stimulating Factor Reduce Stress Oxidative Factors in Parkinson’s Disease, Iranian Biomedical Journal. (2020) 24, no. 2, 89–98, 10.29252/ibj.24.2.89. 31677610 PMC6984711

[9] Lin G. , Wang L. , Marcogliese P. C. , and Bellen H. J. , Sphingolipids in the Pathogenesis of Parkinson’s Disease and Parkinsonism, Trends in Endocrinology and Metabolism. (2019) 30, no. 2, 106–117, 10.1016/j.tem.2018.11.003, 2-s2.0-85057612481. 30528460

[10] Ganapathy K. , Datta I. , Sowmithra S. , Joshi P. , and Bhonde R. , Influence of 6-Hydroxydopamine Toxicity on α-Synuclein Phosphorylation, Resting Vesicle Expression, and Vesicular Dopamine Release, Journal of Cellular Biochemistry. (2016) 117, no. 12, 2719–2736, 10.1002/jcb.25570, 2-s2.0-85028234839. 27064513

[11] Ali N. , Sane M. S. , Tang H. et al., 6-Hydroxydopamine Affects Multiple Pathways to Induce Cytotoxicity in Differentiated LUHMES Dopaminergic Neurons, Neurochemistry International. (2023) 170, 10.1016/j.neuint.2023.105608. 37678429

[12] Mohsenvand Z. , Sameni H. R. , ghanbari A. et al., Neuroprotective Effects of Caffeic Acid Phenethyl Ester Administered With Levodopa and Benserazide in a Rat Model of Parkinson’s Disease, Physiology and Pharmacology. (2025) 29, no. 3, 272–283, 10.61882/phypha.29.3.272.

[13] Cui H. , Elford J. D. , Alitalo O. et al., Nigrostriatal 6-Hydroxydopamine Lesions Increase Alpha-Synuclein Levels and Permeability in Rat Colon, Neurobiology of Aging. (2023) 129, 62–71, 10.1016/j.neurobiolaging.2023.05.007. 37271045

[14] Hernandez-Baltazar D. , Zavala-Flores L. M. , and Villanueva-Olivo A. , The 6-Hydroxydopamine Model and Parkinsonian Pathophysiology: Novel Findings in an Older Model, Neurologia. (2017) 32, no. 8, 533–539, 10.1016/j.nrl.2015.06.011, 2-s2.0-85029036474. 26304655

[15] Zhang L. , Li C. , Zhang Z. et al., DA5-CH and Semaglutide Protect Against Neurodegeneration and Reduce α-Synuclein Levels in the 6-OHDA Parkinson’s Disease Rat Model, Parkinson’s Disease. (2022) 2022, no. 1, 10.1155/2022/1428817. PMC9678466 36419409

[16] Ovallath S. and Sulthana B. , Levodopa: History and Therapeutic Applications, Annals of Indian Academy of Neurology. (2017) 20, no. 3, 185–189, 10.4103/aian.AIAN_241_17, 2-s2.0-85032902868. 28904446 PMC5586109

[17] Tambasco N. , Romoli M. , and Calabresi P. , Levodopa in Parkinson’s Disease: Current Status and Future Developments, Current Neuropharmacology. (2018) 16, no. 8, 1239–1252, 10.2174/1570159x15666170510143821, 2-s2.0-85044071463. 28494719 PMC6187751

[18] Yoosefian M. , Rahmanifar E. , and Etminan N. , Nanocarrier for Levodopa Parkinson Therapeutic Drug; Comprehensive Benserazide Analysis, Artificial Cells, Nanomedicine and Biotechnology. (2018) 46, no. 1, 434–446, 10.1080/21691401.2018.1430583, 2-s2.0-85041225574. 29378432

[19] Su D. , Zhang X. , Su Y. , Chan P. , and Xu E. , Effects of Different Levodopa Doses on Blood Pressure in Older Patients With Early and Middle Stages of Parkinson’s Disease, Heliyon. (2023) 9, no. 7, 10.1016/j.heliyon.2023.e17876. PMC10362309 37483692

[20] Chaudhuri K. R. and Schapira A. H. V. , Non-Motor Symptoms of Parkinson’s Disease: Dopaminergic Pathophysiology and Treatment, The Lancet Neurology. (2009) 8, no. 5, 464–474, 10.1016/S1474-4422(09)70068-7, 2-s2.0-64349083816. 19375664

[21] Carey G. , Görmezoğlu M. , de Jong J. J. A. et al., Neuroimaging of Anxiety in Parkinson’s Disease: A Systematic Review, Movement Disorders. (2021) 36, no. 2, 327–339, 10.1002/mds.28404. 33289195 PMC7984351

[22] Carey G. , Viard R. , Lopes R. et al., Anxiety in Parkinson’s Disease is Associated With Changes in Brain Structural Connectivity, Journal of Parkinson’s Disease. (2023) 13, no. 6, 989–998, 10.3233/jpd-230035. PMC10578283 37599537

[23] Eskow J. K. L. , Angoa-Perez M. , Kuhn D. M. , and Bishop C. , Potential Mechanisms Underlying Anxiety and Depression in Parkinson’s Disease: Consequences of L-DOPA Treatment, Neuroscience & Biobehavioral Reviews. (2011) 35, no. 3, 556–564, 10.1016/j.neubiorev.2010.06.007, 2-s2.0-78649907182. 20615430 PMC2987522

[24] Politis M. and Loane C. , Serotonergic Dysfunction in Parkinson’s Disease and Its Relevance to Disability, The Scientific World Journal. (2011) 11, 1726–1734, 10.1100/2011/172893, 2-s2.0-81455143969. 22125431 PMC3201695

[25] Oertel W. and Schulz J. B. , Current and Experimental Treatments of Parkinson Disease: A Guide for Neuroscientists, Journal of Neurochemistry. (2016) 139, no. S1, 325–337, 10.1111/jnc.13750, 2-s2.0-84991235427. 27577098

[26] Kamińska K. , Lenda T. , Konieczny J. , and Lorenc-Koci E. , Behavioral and Neurochemical Interactions of the Tricyclic Antidepressant Drug Desipramine With L-DOPA in 6-OHDA-Lesioned Rats. Implications for Motor and Psychiatric Functions in Parkinson’s Disease, Psychopharmacology (Berl). (2022) 239, no. 11, 3633–3656, 10.1007/s00213-022-06238-x. 36178508 PMC9584871

[27] Collier T. J. , Srivastava K. R. , Justman C. et al., Nortriptyline Inhibits Aggregation and Neurotoxicity of Alpha-Synuclein by Enhancing Reconfiguration of the Monomeric Form, Neurobiology of Disease. (2017) 106, 191–204, 10.1016/j.nbd.2017.07.007, 2-s2.0-85024400996. 28711409 PMC5793922

[28] Prange S. , Klinger H. , Laurencin C. , Danaila T. , and Thobois S. , Depression in Patients With Parkinson’s Disease: Current Understanding of Its Neurobiology and Implications for Treatment, Drugs & Aging. (2022) 39, no. 6, 417–439, 10.1007/s40266-022-00942-1. 35705848 PMC9200562

[29] Lauterbach E. C. , Repurposing Psychiatric Medicines to Target Activated Microglia in Anxious Mild Cognitive Impairment and Early Parkinson’s Disease, American Journal of Neurodegenerative Disease. (2016) 5, no. 1, 29–51. 27073741 PMC4788730

[30] Peña E. , Mata M. , López-Manzanares L. et al., Antidepressants in Parkinson’s Disease. Recommendations by the Movement Disorder Study Group of the Neurological Association of Madrid, Neurologia. (2016) 10.1016/j.nrl.2016.02.002, 2-s2.0-85006302959. 27004670

[31] Mohammed H. S. , Hosny E. N. , Sawie H. G. , and Khadrawy Y. A. , Transcranial Photobiomodulation Ameliorates Midbrain and Striatum Neurochemical Impairments and Behavioral Deficits in Reserpine-Induced Parkinsonism in Rats, Photochemical and Photobiological Sciences. (2023) 22, no. 12, 2891–2904, 10.1007/s43630-023-00497-z. 37917308

[32] Xing B. , Li Y.-C. , and Gao W.-J. , Norepinephrine Versus Dopamine and Their Interaction in Modulating Synaptic Function in the Prefrontal Cortex, Brain Research. (2016) 1641, 217–233, 10.1016/j.brainres.2016.01.005, 2-s2.0-84961817084. 26790349 PMC4879059

[33] Di Matteo V. , Di Giovanni G. , Pierucci M. , and Esposito E. , Serotonin Control of Central Dopaminergic Function: Focus on In Vivo Microdialysis Studies, Progress in Brain Research. (2008) 172, 7–44, 10.1016/s0079-6123(08)00902-3, 2-s2.0-50649083263. 18772026

[34] Berends H. I. , Nijlant J. M. , Movig K. L. , Van Putten M. J. , Jannink M. J. , and Ijzerman M. J. , The Clinical Use of Drugs Influencing Neurotransmitters in the Brain to Promote Motor Recovery After Stroke; A Cochrane Systematic Review, European Journal of Physical and Rehabilitation Medicine. (2009) 45, no. 4, 621–630. 20032921

[35] Council NR , Guide for the Care and Use of Laboratory Animals: Eighth Edition, 2011, The National Academies Press. 21595115

[36] Bis-Humbert C. , García-Cabrerizo R. , and García-Fuster M. J. , Dose-Dependent Opposite Effects of Nortriptyline on Affective-Like Behavior in Adolescent Rats: Comparison With Adult Rats, European Journal of Pharmacology. (2021) 910, 10.1016/j.ejphar.2021.174465. 34464602

[37] Safari M. , Jafari B. , Zarbakhsh S. et al., G-CSF for Mobilizing Transplanted Bone Marrow Stem Cells in Rat Model of Parkinson’s Disease, Iranian Journal of Basic Medical Sciences. (2016) 19, no. 12, 1318–1324, 10.22038/ijbms.2016.7918, 2-s2.0-85004125564. 28096964 PMC5220237

[38] Jadidi T. , Asadian N. , Jadidi M. , Safari M. , Sameni H. R. , and Semnani V. , Electromagnetic Field Exposed Stem Cells Repaired Parkinson’s Disease Symptoms in a Rat Model, International Journal of Radiation Research. (2023) 21, no. 1, 61–66, 10.52547/ijrr.21.1.8.

[39] Slézia A. , Hegedüs P. , Rusina E. et al., Behavioral, Neural and Ultrastructural Alterations in a Graded-Dose 6-OHDA Mouse Model of Early-Stage Parkinson’s disease, Scientific Reports. (2023) 13, no. 1, 10.1038/s41598-023-46576-0. PMC10636184 37945922

[40] Rosa I. , Di Censo D. , Ranieri B. et al., Comparison Between Tail Suspension Swing Test and Standard Rotation Test in Revealing Early Motor Behavioral Changes and Neurodegeneration in 6-OHDA Hemiparkinsonian Rats, International Journal of Molecular Sciences. (2020) 21, no. 8, 10.3390/ijms21082874. PMC7216013 32326015

[41] Omotoso G. , Oloyede O. , Lawal S. et al., Permethrin Exposure Affects Neurobehavior and Cellular Characterization in Rats’ Brain, nvironmental Analysis Health and Toxicology. (2020) 35, no. 4, 10.5620/eaht.2020022. PMC7829406 33434422

[42] Liu X. L. , Fan L. , Yue B. H. , and Lou Z. , Saikosaponin A Mitigates the Progression of Parkinson’s Disease Via Attenuating Microglial Neuroinflammation Through TLR4/MyD88/NF-κB Pathway, European Review for Medical and Pharmacological Sciences. (2023) 27, no. 15, 6956–6971, 10.26355/eurrev_202308_33268. 37606106

[43] Bayram-Weston Z. , Olsen E. , Harrison D. J. , Dunnett S. B. , and Brooks S. P. , Optimising Golgi-Cox Staining for Use With Perfusion-Fixed Brain Tissue Validated in the zQ175 Mouse Model of Huntington’s Disease, Journal of Neuroscience Methods. (2016) 265, 81–88, 10.1016/j.jneumeth.2015.09.033, 2-s2.0-84973160119. 26459195 PMC4863524

[44] Gorgich E. A. C. , Parsaie H. , Yarmand S. , Baharvand F. , and Sarbishegi M. , Long-Term Administration of Metformin Ameliorates Age-Dependent Oxidative Stress and Cognitive Function in Rats, Behavioural Brain Research. (2021) 410, 10.1016/j.bbr.2021.113343. 33965434

[45] Reeve A. , Simcox E. , and Turnbull D. , Ageing and Parkinson’s Disease: Why is Advancing Age the Biggest Risk Factor?, Ageing Research Reviews. (2014) 14, no. 100, 19–30, 10.1016/j.arr.2014.01.004, 2-s2.0-84894310713. 24503004 PMC3989046

[46] Qiu H. , Liu C. , and Wang Z. , Levodopa-Induced Motor Complications Associated With Benserazide and Carbidopa in Parkinson’s Disease: A Disproportionality Analysis of the FAERS Database, Frontiers in Pharmacology. (2025) 16, 10.3389/fphar.2025.1529932. PMC11922873 40115262

[47] Ezzedin M. , Safari M. , Zarbakhsh S. et al., The Effect of Co-Administration of Levodopa, Benserazide and Nortriptyline of Behavioral Symptoms in an Exprimental Model of Parkinson’s Rats, Research Square. (2022) 10.21203/rs.3.rs-2097021/v1.

[48] Van Camp N. , Vreys R. , Van Laere K. et al., Morphologic and Functional Changes in the Unilateral 6-Hydroxydopamine Lesion Rat Model for Parkinson’s Disease Discerned With microSPECT and Quantitative MRI, Magma. (2010) 23, no. 2, 65–75, 10.1007/s10334-010-0198-7, 2-s2.0-77952423316. 20169465

[49] Menza M. , Dobkin R. D. , Marin H. et al., A Controlled Trial of Antidepressants in Patients With Parkinson Disease and Depression, Neurology. (2009) 72, no. 10, 886–892, 10.1212/01.wnl.0000336340.89821.b3, 2-s2.0-64049105010. 19092112 PMC2677475

[50] Dobkin R. D. , Menza M. , Bienfait K. L. et al., The Impact of Antidepressant Treatment on Cognitive Functioning in Depressed Patients With Parkinson’s Disease, Journal of Neuropsychiatry and Clinical Neurosciences. (2010) 22, no. 2, 188–195, 10.1176/jnp.2010.22.2.188, 2-s2.0-77952394139. 20463113 PMC2872099

[51] Kim B. , Weerasinghe-Mudiyanselage P. D. E. , Ang M. J. et al., Changes in the Neuronal Architecture of the Hippocampus in a 6-Hydroxydopamine-Lesioned Rat Model of Parkinson Disease, International Neurourology Journal. (2022) 26, no. 2, S94–S105, 10.5213/inj.2244252.126. 36503212 PMC9767684

[52] Fanselow M. S. and Dong H. W. , Are the Dorsal and Ventral Hippocampus Functionally Distinct Structures?, Neuron. (2010) 65, no. 1, 7–19, 10.1016/j.neuron.2009.11.031, 2-s2.0-73549123839. 20152109 PMC2822727

[53] Li X. , Du Z. J. , Xu J. N. et al., mGluR5 in Hippocampal CA1 Pyramidal Neurons Mediates Stress-Induced Anxiety-Like Behavior, Neuropsychopharmacology. (2023) 48, no. 8, 1164–1174, 10.1038/s41386-023-01548-w. 36797374 PMC10267178

[54] Wang X. , Pal R. , Chen X. W. , Limpeanchob N. , Kumar K. N. , and Michaelis E. K. , High Intrinsic Oxidative Stress May Underlie Selective Vulnerability of the Hippocampal CA1 Region, Molecular Brain Research. (2005) 140, no. 1-2, 120–126, 10.1016/j.molbrainres.2005.07.018, 2-s2.0-26844581733. 16137784

