# Supplementary material for: Synergistic Effects of Levodopa, Benserazide, and Nortriptyline on Behavioral Impairments and Brain Pathology in an Experimental Rat Model of Parkinson’s Disease
Source: Neurol Res Int. 2026 Jan 28;2026:9986180. doi: 10.1155/nri/9986180 (PMC12852062; doi:10.1155/nri/9986180)
Supplement: Supplementary file 1 — Supporting Information Additional supporting information can be found online in the Supporting Information section. [file NRI-2026-9986180-s001.zip › Graphical abstract.docx]

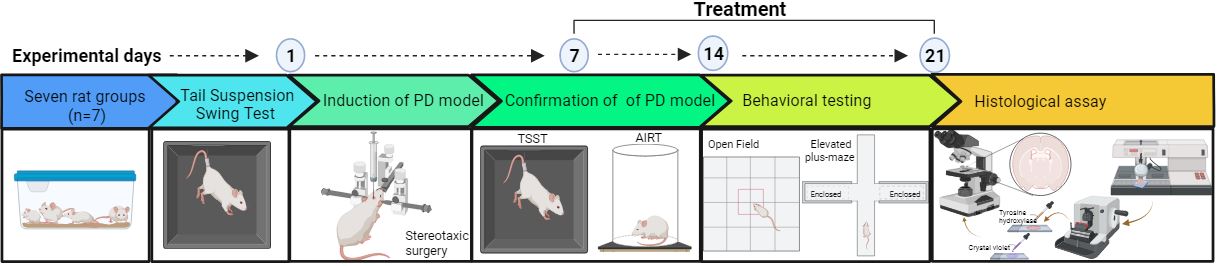


Graphical abstract

The main stages of the research project, including induction of the PD model, treatment duration, behavioural and histological studies are briefly shown in the graphical abstract.
