# Supplementary material for: Synergistic Effects of Levodopa, Benserazide, and Nortriptyline on Behavioral Impairments and Brain Pathology in an Experimental Rat Model of Parkinson’s Disease
Source: Neurol Res Int. 2026 Jan 28;2026:9986180. doi: 10.1155/nri/9986180 (PMC12852062; doi:10.1155/nri/9986180)
Supplement: Supplementary file 1 — Supporting Information Additional supporting information can be found online in the Supporting Information section. [file NRI-2026-9986180-s001.zip › Highlights.docx]

**Highlights**

1. In addition to improving anxiety and stress-like behaviors in PD, nortriptyline may also help improve motor activity.
2. The best results of improving behavioral and motor performance were associated with the 10 mg/kg dose in the co-administration of 5, 10, and 20 mg/kg of nortriptyline with levodopa and benserazide.
3. To reduce the side effects of levodopa in the treatment of PD, the use of nortriptyline is recommended.
4. The use of higher doses of nortriptyline in the treatment of PD symptoms requires more caution due to the existence of opposite results.
5. The nortriptyline can protect neurons in the hippocampal C1 area by reducing levels of oxidative stress.
